# Application of metabolomics in osteoporosis research

**DOI:** 10.3389/fendo.2022.993253

**Published:** 2022-11-14

**Authors:** Zhenyu Zhao, Zhengwei Cai, Aopan Chen, Ming Cai, Kai Yang

**Affiliations:** ^1^ Department of Orthopaedics, Shanghai Tenth People’s Hospital, School of Medicine, Tongji University, Shanghai, China; ^2^ Shanghai Key Laboratory for Prevention and Treatment of Bone and Joint Diseases, Shanghai Institute of Traumatology and Orthopaedics, Ruijin Hospital, Shanghai Jiao Tong University School of Medicine, Shanghai, China

**Keywords:** metabolomics, osteoporosis, pathogenic mechanism, herbal medicine, treatment

## Abstract

Osteoporosis (OP) is a systemic disease characterized by bone metabolism imbalance and bone microstructure destruction, which causes serious social and economic burden. At present, the diagnosis and treatment of OP mainly rely on imaging combined with drugs. However, the existing pathogenic mechanisms, diagnosis and treatment strategies for OP are not clear and effective enough, and the disease progression that cannot reflect OP further restricts its effective treatment. The application of metabolomics has facilitated the study of OP, further exploring the mechanism and behavior of bone cells, prevention, and treatment of the disease from various metabolic perspectives, finally realizing the possibility of a holistic approach. In this review, we focus on the application of metabolomics in OP research, especially the newer systematic application of metabolomics and treatment with herbal medicine and their extracts. In addition, the prospects of clinical transformation in related fields are also discussed. The aim of this study is to highlight the use of metabolomics in OP research, especially in exploring the pathogenesis of OP and the therapeutic mechanisms of natural herbal medicine, for the benefit of interdisciplinary researchers including clinicians, biologists, and materials engineers.

## 1. Introduction

OP is a systemic metabolic bone disease characterized by damage to the microstructure of bone tissue and reduced bone mass, resulting in increased bone fragility and susceptibility to fractures. The main clinical manifestations of OP are height loss and hunchback, which increases the risk of fractures in multiple parts such as the hip and spine, and the probability of fracture varies from country to country (1–3). Fractures are predicted to occur in women over age 50 and in one in five men, resulting in limited quality of life and increased morbidity and mortality (4–6). Therefore, with the increasing aging of the population, complications such as osteoporotic fractures have become a worldwide problem, and the economic burden is increasing exponentially. Therefore, it is particularly important to comprehensively prevent and treat OP from the aspects of pathogenic mechanism, prevention, and treatment.

At present, OP has received great attention and extensive research, and a routine prevention and treatment system has also been formed in clinical practice (7–11). For instance, for the prevention of OP, patients are mainly encouraged to take calcium and vitamin D supplements early in clinical practice, and increase the time of sunlight exposure. However, this method has poor specificity and effectiveness, and lacks pertinence and targeting, making it difficult to effectively slow down the development of bone loss. At the same time, dual energy X-ray is insufficient in revealing the strength and structure of the bone tissue (12, 13). Although various drugs are developed in clinical OP treatment *via* confirmed mechanisms, including hormone replacement, alendronate sodium, parathyroid hormone, and RANKL inhibitors, etc. (14–16), the osteoporotic symptom still hard to completely reverse. Therefore, more therapeutic clues, especially metabolic ones should be concerned in the field of OP prevention.

The collection of small-molecule chemical entities involved in metabolic forms the metabolome. Metabolomics has been redefined from a simple biomarker identification tool to a technique for discovering active drivers of biological processes (17–19). It detects multiple metabolite changes during environmental exposure in a high-throughput form and are closely related to pathological phenotype, especially for multifunctional disease such as OP (20, 21). Therefore, metabolomics emerges an increasingly important role in the systematic study of OP. It is worth noting that, using metabolomics, the functions of traditional Chinese medicines such as Epimedium, Gushudan on OP treatment have been explored. However, there is a lack of systematic induction and research on the metabolic mechanism of various natural herbs in the treatment of OP.

Thus, metabolomics plays an important role in multi-field research of OP, which can deeply explore many problems closely related to OP and provide a new approach for comprehensive research and evaluation of OP. This review systematized various applications of metabolomics in OP research, including mechanism exploration, prevention, prediction, and drug treatment effect. In particular, we focused on the key metabolite changes in OP and after treatment with natural herbal medicines. Lots of metabolites are summarized to correlated with OP treatment, which could be useful for clinical transformation in related fields.

## 2. Abnormal metabolism in OP

Bone undergoes a constantly active metabolic cycle which is essential to maintain a healthy bone composition through the deposition and absorption of bone matrix and minerals. Imbalance and/or dysregulation of specific biochemical cascades of enzymes involved in protein, fat, and carbohydrates in bone metabolism can lead to various types of osteocyte dysfunction and related metabolic bone disease (22, 23).

Postmenopausal OP is characterized by loss of estrogen that leads to metabolic disorders in bone tissue. Metabolomics assays are factors associated with biological or metabolic status, and these metabolites are highly correlated pathogenic mechanisms of postmenopausal OP (24–31). On the other hand, abnormal differentiation of bone mesenchymal stem cells is related to the occurrence of senile OP. What’s more, under the influence of endogenous and exogenous factors such as hormone abuse, menopause, and aging, BMS over-differentiate into fat cells instead of osteoblasts, which often leads to bone metabolism imbalance and even OP. Therefore, a comprehensive understanding of the cellular metabolism and functional changes of bone marrow mesenchymal stem cells with aging is of great significance for the mechanism exploration and clinical treatment of senile OP.

There is increasing evidence that some of the causative factors are modifiable risk factors for OP, such as abnormal drug intake, high fat, and abnormal hormone levels. Studies have shown that these substances can induce secondary OP by regulating changes in metabolite levels (32–38).

Cholesterol participates in many cellular structures, and hydroxysterols, steroid hormones and bile acids play an important role in the formation of cell membranes. Therefore, dysfunction in cholesterol synthesis associates with various diseases and disorders (39, 40). In the OP model, the precise control of cholesterol synthesis and transport is affected, evidenced by the abnormal level of isoprene and squalene. Fatty acid metabolism involves a series of enzymes that degrade fatty acids into bioactive substrates to synthesize straight chain fatty acids, which are stored in adipose tissue as triglycerides (41, 42). In postmenopausal OP, fatty acyl-coa and pyruvate are converted to acetyl-coa by glycolysis, and subsequent metabolic pathways for synthesis of NADH, guanosine triphosphate and amino acids are disrupted (42, 43). Glycogen is easily mobilized as a long-branched polymer of glucose residues and can be broken down into glucose to provide the body with the required energy. Human osteoblasts and bone marrow mesenchymal stem cells in patients with secondary OP can be manifested as abnormal glucose metabolism of creatine, glucokinase and phosphorylase (44).

Therefore, in recent years, it has been found that there are many metabolic pathways in OP, related with abnormal bone remodeling. However, the changes of these metabolites and pathways and their roles in the pathogenesis of OP remain unclear (44, 45).

## 3. Metabolomics sample preparation and platform technologies

### 3.1 Metabolomics sample preparation

OP can be classified as primary or secondary according to its cause. For primary OP, biological samples can be collected from postmenopausal women and the elderly in clinical studies, whereas animal models could be accomplished by surgical ovaries resection (46, 47). For secondary OP, most samples are come from animal models, including glucocorticoids injection, fixation, special diet and retinoic acid lavage (45).

In sample preparation for metabolomics of OP, the following samples are usually included: urine, plasma, serum, osteocytes, bone cells, etc (48, 49). Urine samples are usually centrifuged directly without any dilution treatment, or diluted with pure water. Protein-rich serum and plasma are deproteinized with organic solvents such as acetonitrile and methanol. In metabolomics analysis, plasma and serum samples also require the use of silylation reagents such as trifluoroacetamide and trimethylsilane to improve the stability of metabolites. Proteins in blood samples are ultrafiltered through high molecular weight cut-off filters. The pH of the sample has a significant effect on the chemical shifts observed in NMR spectroscopy, so it is important to control the pH of the biofluid sample. To provide a stable environment for urine samples, a phosphate buffer (pH 6.8) is usually used (50–52).

For osteoblast samples, the cell pellet was resuspended in water, and then the membrane was broken with ultrasound and extracted with cold water mixed with methanol (53). After the above extraction process, samples are diluted or centrifuged in mobile phase, evaporated to dryness, and finally resuspended in a compatible solvent for further injection into metabolomics-related systems (17, 54). To obtain accurate metabolites, cells need to stop their metabolic activity almost immediately, and the classical methods include enzymatic denaturation and freezing (55). After extraction of metabolites from bone tissue samples, the tissue is pressed between metal plates in the presence of liquid nitrogen for rapid collection and rapid freezing of bone tissue. Care must be taken before and during sampling to avoid metabolic changes, which may be caused by anesthesia and other procedures (56).

### 3.2 Metabolomics platform technologies

After preparing the relevant samples, it is important to choose a suitable protocol and platform technologies in exploring the metabolomics. Currently, LC/MS (liquid chromatography/MS), GC/MS (gas chromatography/MS) and 1H NMR (nuclear magnetic resonance) are the main tools for exploring OP metabolomics (1, 57, 58). 1H NMR is suitable for the preliminary exploration of metabolomics, where most compounds can be detected. This method does not damage the sample, pre-treatment is relatively simple and can be used for quantitative data analysis. At now, 1H NMR still plays an important role in metabolomics because of its high ability in detecting the metabolites and elucidate structures *in vivo*. Its disadvantage is the narrow detection window and lack of sensitivity (59, 60).

MS has great advantages over 1H NMR in terms of sensitivity and specificity, and MS can detect potential new biomarkers associated with OP and has a good ability to detect metabolites at low concentrations and without parental interference, which, combined with modern high-resolution MS, plays an important role in the study of metabolomics. However, it also has the disadvantage of poor homogeneity and integrity (61, 62). Chromatography can be used for the separation of complex osteoporotic compounds and has promising applications. Another advantage of chromatography is the possibility of separating isomers. The addition of chromatography can improve the detection of low concentrations of metabolites, increase the coverage of metabolomics, and improve the quantitative accuracy of MS, but it also has disadvantages, such as insufficient qualitative capabilities. There are many kinds of chromatographic derivatives, such as reversed-phase liquid chromatography (RPLC), high performance liquid chromatography (HPLC), reversed-phase liquid chromatography (RPLC) hydrophilic liquid chromatography (HILC), and ultra-high energy liquid chromatography (UHPLC) (54). Currently, different chromatographic methods are often combined with MS to complement each other. It is often used in combination with GC/MS and LC/MS and has a wide range of applications in exploring OP with high sensitivity and good selectivity. It also allows the quantitative and qualitative analysis of complex metabolic compounds (57, 63, 64).

## 4. Metabolomics in OP pathogenesis research

**Figure 1:**
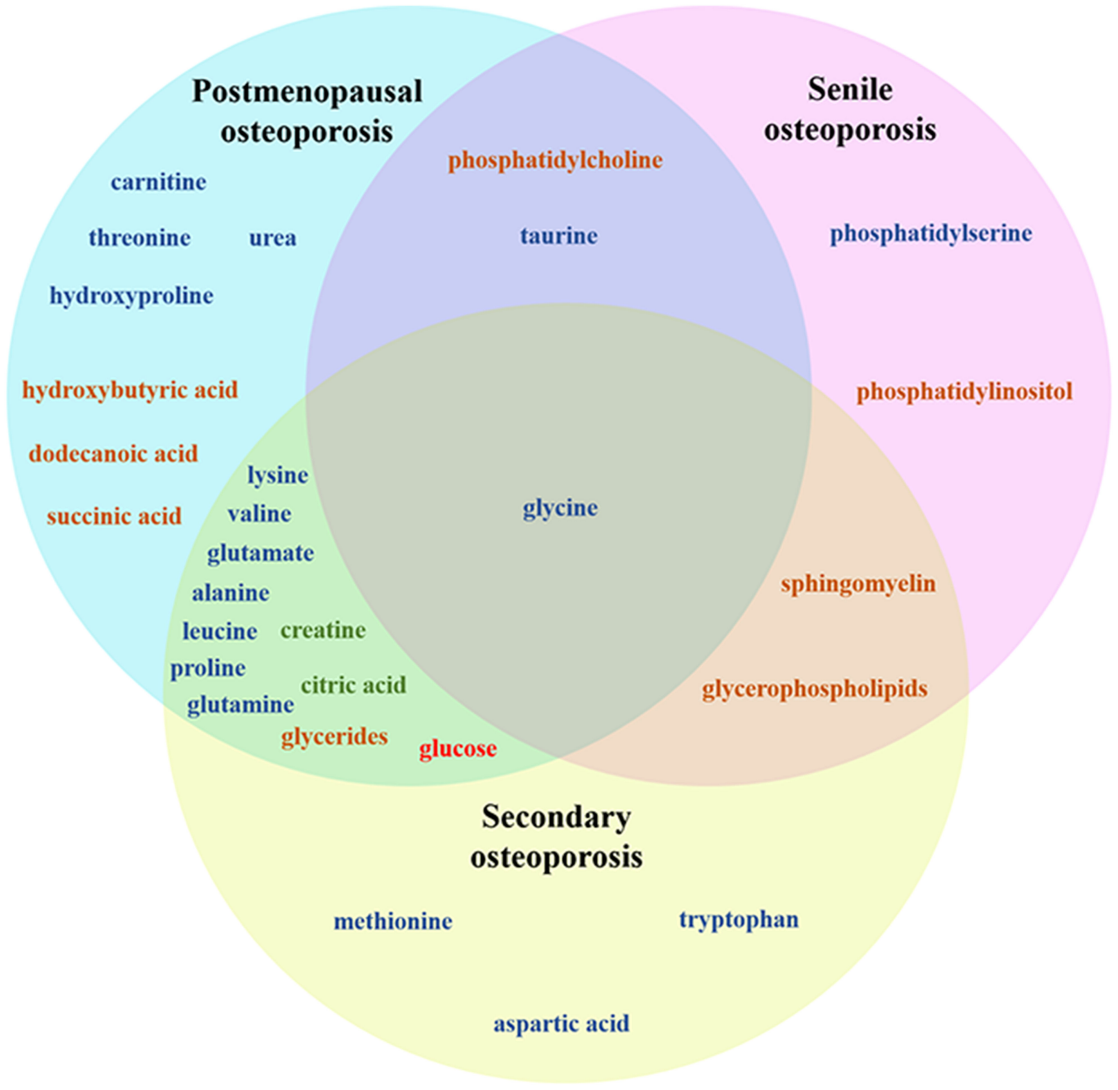
Venn diagram of metabolite changes in different types of OP pathogenesis. Dark blue: Amino acid metabolism product, dark yellow: Lipid metabolism product, dark green: Energy metabolism product, red: sugar metabolite.

In recent years, using metabolomics, the pathogenesis of OP has been comprehensively studied. OP can lead to profound metabolic changes in bone marrow and bone, involving many different metabolites and metabolic pathways (65, 66), as shown in **Figure 1**. The related mechanisms mainly involve amino acid metabolism, lipid metabolism, glucose metabolism, energy metabolism, etc. Lipid metabolism plays an important role in the pathogenesis of senile OP. In addition, lipid metabolism in idiopathic OP is also disturbed. Secondary OP has a clear etiology, and its metabolization-related pathogenesis varies from disease to disease, usually involving lipid metabolism, amino acid metabolism, mitochondrial energy metabolism, etc. (24–38, 67, 68), as shown in **Table 1**.

**Table 1:** The application of metabolomics in exploring the pathogenic mechanism of various types of OP.

Types of OP	Sample type	Study size	Analyticalmethod	Key metabolic mechanism pathways	References
postmenopausal OP	patient serum	571	LC-MS	amino acid metabolism	(29)
	patient serum	1193	CE-TOFMS	energy metabolism, amino acid metabolism	(31)
	patient serum	499	LC-MS	lipid metabolism, phenylpropionic acid metabolism and bile acid metabolism	(28)
	patient stool	108	LC-MS	amino acid metabolism, nucleotide metabolism	(26)
	patient serum	517	LC-MS	fatty acid metabolism	(25)
	patient serum	631	Not mentioned	amino acid metabolism, adrenal androgen metabolism	(24)
	rat bone tissue	18	GC-MS	amino acid metabolism, purine metabolism, fatty acid metabolism	(30)
	rat serum	14	LC-MS	bile acid metabolism	(27)
	patient serum	1552	LC-MS	amino acid metabolism, lipid metabolism	(69)
	patient serum	97	LC-MS	amino acid metabolism, lipid metabolism, glucose metabolism	(70)
	patient serum	109	LC-MS	lipid metabolism, sugar metabolism, amino acid metabolism, nucleic acid metabolism	(48)
	patient urine	322	GC-MS	energy metabolism, amino acid metabolism, glucose metabolism	(71)
	patient serum	364	GC-MS	lipid metabolism, amino acid metabolism	(72)
Senile OP	cell culture	cells at 90% density	UPLC−MS	lipid metabolism, amino acid metabolism	(67)
	cell culture	Not mentioned	MS-MS	lipid metabolism, amino acid metabolism	(68)
	patient serum	729	LC-MS	amino acid metabolism, energy metabolism	(73)
	patient serum	69	LC-MS	amino acid metabolism, lipid metabolism	(74)
Secondary OP	patient serum	18	1H NMR	energy metabolism, amino acid metabolism, glucose metabolism	(75)
	patient serum	1545	UHPLC-MS	fat metabolisim	(32)
	patient serum	119	LC-MS	energy metabolism, amino acid metabolism	(33)
	laying hen serum	88	Not mentioned	lipid metabolism, amino acid metabolism	(34)
	mouse serum	12	UHPLC-MS/MS	purine metabolism, lipid metabolism	(35)
	goat serum	28	LC–MS	amino acid metabolism, lipid metabolism	(38)

### 4.1 Different types of metabolism

#### 4.1.1 Amino acid metabolism

The metabolism of amino acids in the body includes two aspects. On the one hand, it is mainly used to synthesize proteins, polypeptides, and other nitrogen-containing substances unique to the body itself. In addition, amino acids can be decomposed into amines, α-keto acids, and carbon dioxide through a series of combined deamination, deamination, decarboxylation, and transamination effects. These carbon dioxide, alpha-keto acids and amines can be converted into lipids, non-essential amino acids, and sugars, and can also be oxidized to release energy using the tricarboxylic acid cycle, while producing water and carbon dioxide. Therefore, amino acid metabolism plays an important role in the pathogenesis of OP. Some studies have consistently demonstrated that some substances in amino acid metabolism might related with the pathogenesis of OP. Scientist studied in the metabolism of middle-aged Japanese women showed that lysine was correlated with the menopausal status of women, and increased gradually with the change of premenopausal, perimenopausal and postmenopausal (31). In addition, the effects of glucocorticoid-induced short-and long-term OP on lipids and plasma metabolites was investigated in ovariectomized sheep. Lysine was also found to distinguish between normal and low bone mineral density (BMD) groups (38). What’s more, a GC-MS analysis in metabolic profiles of postmenopausal OP progression in 364 Chinese women reflected the epochal changes of lysine in the pathogenesis of OP (72).

Novel metabolite changed in middle-aged Japanese women were studied. The study found that carnitine was associated with women’s menopausal status and gradually increased with the change of menopause (31). The metabolites and metabolic pathways associated with changes in BMD in postmenopausal and perimenopausal women with OP was systematically investigated. Carnitine significantly affects changes in BMD (25). In addition, patient serum samples are efficiently analyzed by metabolomics using untargeted MS. The study found that compared with the control group, the carnitine content of the OP group was significantly imbalanced (74). These studies would help to establish that the pathogenic mechanism of healthy bones and OP was closely related to metabolite carnitine.

Metabolomics techniques were used to discover differences in metabolites of bone metabolic disorders between healthy volunteers and osteoporotic patients. Abnormal metabolism of valine might serve as a key mechanism of OP (31, 38, 75). The relationship between OP and amino acid metabolism was further explored. The results of the study indicate that the change of the metabolite glutamate may play an important role in the pathogenesis of OP (26, 36, 70).

In addition, some recent studies have also suggested that the following amino acids may be closely related to the pathogenesis of OP, including: alanine (30, 36, 75), tryptophan (34, 38, 72), methionine (36, 38, 70), phosphatidylserine (67, 68), urea (28, 30), glycine (69, 73, 75), threonine (69, 70), leucine (69, 75), proline (31, 70, 75), aspartic acid (36, 75), hydroxyproline (31, 72), taurine (31, 71, 73), glutamine (31, 75), as shown in **Table 2**.

**Table 2:** Metabolomics is used to explore mechanisms of OP, with the same metabolites found in different studies.

Type of metabolism	Co-discovered metabolites	Types of OP	Sample type	References
Amino acid metabolism	lysine	postmenopausal OP, secondary OP	patient serum, goat serum	(31, 38, 72)
	carnitine	postmenopausal OP	patient serum	(25, 31, 74)
	valine	postmenopausal OP, secondary OP	patient serum, goat serum	(31, 38, 75)
	glutamate	postmenopausal OP, secondary OP	patient stool	(26, 36)
	alanine	secondary OP, postmenopausal OP	rat bone tissue	(30, 36, 75)
	tryptophan	secondary OP	laying hen serum, goat serum	(34, 38, 72)
	methionine	secondary OP	goat serum	(36, 38, 70)
	phosphatidylserine	senile OP	cell culture	(67, 68)
	urea	postmenopausal OP	rat bone tissue, patient serum	(28, 30)
	glycine	postmenopausal OP, senile OP secondary OP	patient serum	(69, 73, 75)
	threonine	postmenopausal OP	patient serum	(69, 70)
	leucine	postmenopausal OP, secondary OP	patient serum	(69, 75)
	proline	postmenopausal OP, secondary OP	patient serum	(31, 70, 75)
	aspartic acid	secondary OP	patient serum, patient stool	(36, 75)
	hydroxyproline	postmenopausal OP	patient serum	(31, 72)
	taurine	postmenopausal OP, senile OP	patient serum	(31, 71, 73)
	glutamine	postmenopausal OP, secondary OP	patient serum	(31, 75)
Lipid metabolism	dodecanoic acid	postmenopausal OP	patient serum	(25, 28)
	hydroxybutyric acid	postmenopausal OP	rat bone tissue, patient serum	(25, 28, 30)
	sphingomyelin	senile OP, secondary OP	cell culture, patient serum	(32, 67, 69, 70) (74)
	phosphatidylinositol	senile OP	cell culture	(67, 68)
	glycerophospholipids	senile OP, secondary OP	cell culture, patient serum	(32, 68, 70)
	phosphatidylcholine	postmenopausal OP, senile OP	patient serum	(67, 69, 74)
	glycerides	postmenopausal OP, secondary OP	patient serum	(32, 70)
	succinic acid	postmenopausal OP	patient serum	(31, 71)
Energy metabolism	creatine	postmenopausal OP, secondary OP	rat serum, patient serum	(27, 69, 75)
	citric acid	postmenopausal OP, secondary OP	patient serum, patient urine	(48, 71, 75)
Glucose metabolism	glucose	postmenopausal OP, secondary OP	patient serum	(28, 71, 75)

#### 4.1.2 Lipid metabolism

Lipids are an important material basis for maintaining cell function and cell proliferation. Several studies have found that some lipid metabolites have been found many times in various samples of OP, which might play an important role in the pathogenesis of OP.

Senescence-related lipid metabolism might play an important role in the abnormal differentiation of BMSCs. The declining trend of sphingomyelin describes lipid responses that might lead to abnormal differentiation of bone marrow-derived mesenchymal stem cells during aging (67). Mendelian randomization analysis showed that sphingomyelin was inversely associated with BMD (32). In Singaporean Chinese postmenopausal women, the association between blood lipids and femoral neck BMD was explored using metabolomics. There was a significant correlation between sphingomyelin and BMD reduction (69). In addition, other studies have also explored the association between BMD and sphingomyelin, indicating that sphingomyelin plays a key role in the pathogenesis of OP (70, 74).

Metabolomics with OP bone marrow and bone also showed that hydroxybutyric acid biosynthesis was disturbed. Assessment of differential metabolites improves understanding of metabolic relationships between kidney-bone axis and tissues in ovariectomized rats. Using a metabolomic approach, serum samples from early menopausal and perimenopausal women were analyzed. These results suggested that hydroxybutyric acid might play a role in the mechanism of osteoporotic bone remodeling (25, 28, 30).

The lipidomic strategy was used to observe the expression of related enzymes and lipids in the membranes of MSCs of different ages and proliferation states. Several studies have found that the changes of glycerophospholipids are closely related to the metabolic function mechanism mediated by bone marrow mesenchymal stem cells (32, 68, 70). Serum samples were analyzed using an untargeted MS-based metabolomic approach. Phosphatidylcholine metabolites were significantly dysregulated in the OP group compared with the control group. This metabolome will contribute to the study of disease mechanisms that promote bone health and OP progression (67, 69, 74).

In addition, other lipid metabolites, such as Glycerides (32, 70), succinic acid (31, 71), dodecanoic acid (37, 40), phosphatidylinositol (67, 68), etc., have been proved by many studies to be closely related to the pathogenesis of OP, as shown in **Table 2**.

#### 4.1.3 Other metabolism

In recent years, the pathogenesis of OP has been continuously explored by means of metabolomics, and some key metabolites of energy metabolism and glucose metabolism also play an important role in the mechanism of OP.

A postmenopausal OP mouse model was used to compare metabolome changes in the control and OP groups. Metabolites creatine was significantly different (27). The pathogenesis of OP is revealed from the perspective of microbe-gut-metabolic bone axis regulation, which provided a new entry point for the pathogenesis of OP. OP-related metabolomic markers were examined to reveal underlying mechanisms of OP. Creatine has changed significantly (69). In addition, metabolomic techniques were used to discover differences in metabolites of bone metabolic disorders between healthy volunteers and diabetic patients. Changes in creatine levels were also found (75). Therefore, the metabolic abnormalities of creatine might serve as a key substance in the underlying pathogenic mechanism of OP.

Metabolites with significant differences between estrogen levels and BMD. Metabolite citric acid changes were useful markers of bone loss and/or estrogen deficiency (48). Metabolomics techniques were used to discover differences in metabolites of bone metabolic disorders between healthy volunteers and diabetic patients. Citric acid level was also significantly changed (75). This metabolite abnormality could be used as a key indicator of the pathogenesis of diabetic OP. Pathological features of postmenopausal OP were revealed, and metabolic pathways and biomarkers that might be associated with OP were explored. citric acid was also found to be a potential biomarker of OP (71). Therefore, citric acid was related to the pathogenesis of OP.

Using metabolomic profiling methods, metabolic alterations in postmenopausal women and elderly OP compared with healthy people were analyzed. These studies all found that glucose played a role in the mechanism of osteoporotic bone remodeling (28, 71, 75).

OP is a classic age-related disease that is often considered a “silent disease” because there are no symptoms for many years before a fracture occurs. Therefore, it is of great practical significance to deeply study the pathogenic mechanism of OP from the perspective of metabolism, which can further promote the early prevention, diagnosis, and intervention of OP from the perspective of mechanism (1–3). To sum up, many studies have shown that OP will experience various metabolite changes in various stages of the disease, including amino acid metabolism, lipid metabolism and energy metabolism (40, 69, 71). These different studies all found some of the same metabolites for the above metabolic pathways. Therefore, these same metabolites played important roles in the pathogenesis of OP, and future monitoring of changes in these metabolites by metabolomics is important to achieve further research in OP.

### 4.2 Factors associated with the metabolomic outcome of OP

OP is a heterogeneous disease. Therefore, vitamin D, diabetes, race, and other factors should be considered when studying OP using metabolomic approaches. Vitamin D inhibits osteoclast recruitment, prevents estrogen deficiency, and enhances osteoblast precursor cell proliferation and osteoblast activity (76). A series of studies found that vitamin D levels can significantly alter amino acids, energy metabolism, levels of sugars and their derivatives, and organic acids in patients with OP, thus affecting the metabolomic outcome of OP (77, 78). In addition, different blood calcium levels affected the metabolism of lipids and amino acids such as taurine, glycerophospholipids and glycine, thus causing changes in the metabolomic outcome of OP, which ultimately affected BMD and bone degeneration (79, 80). Recent studies have found that temperature increases the total amount of polyamines in the body, and that inhibition of polyamine biosynthesis in the body limited the protective effect on bone (81).

Severe metabolic disturbances in diabetes can lead to OP. Findings suggest that diabetes mellitus combined with OP will further lead to significant changes in amino acid metabolism and energy metabolism, such as tricarboxylic acid cycle products and various branched-chain amino acids (75). OP combined with osteoarthritis will further alter the phospholipid precursors, energy metabolism and amino acid metabolites of OP (82). In addition, lipoprotein and amino acid metabolites are significantly different when OP is associated with atherosclerosis (83).

Ethnic differences are also important factors influencing metabolomic outcomes in OP. The association between lipid and amino acid metabolites and BMD changes was significant in Asian women with OP in China and Singapore. In particular, dodecanoic acid played an important role in metabolites (25, 70). However, TwinsUK-based studies have mainly identified amino acid and hormone metabolites and found a causal relationship between them and BMD. Lipid metabolites and other amino acid metabolites were different from those in Asians (24). In addition, these studies have shown that the severity of OP varies among ethnic groups. Therefore, the changes in the levels of osteoporotic metabolites and the occurrence and development of OP explored by metabolomics are different based on different races (24, 25, 70).

As a secondary OP, OP caused by intestinal flora has attracted more and more attention in recent years. The microorganisms in the gastrointestinal tract are collectively referred to as the gut flora and consist of approximately 10 trillion bacteria (84). Recent studies have provided substantial evidence for the existence of a “gut microbiota-metabolite-bone axis”, and OP is closely associated with the development and progression of gut microbiota imbalances (26, 27, 35, 85–87). He et al (26) combined LC-MS metabolomics with 16S rRNA gene sequencing. The results showed that mutations in gut bacteria such as Klebsiella and Clostridium interfered with changes in the metabolic levels of acetylmannosamine, type I collagen C-terminal peptide and collagenogenic peptide, and mediated postmenopausal bone loss. Other studies have found that OP was associated with the functional, taxonomic and β-diversity composition of the gut microbiota. Oscillating bacteria, Brucella, Actinomyces and other intestinal flora acted mainly on the metabolism of tryptophan and tyrosine and the degradation of isoleucine, leucine and valine, thus negatively regulating BMD in OP (86). Wen et al. (27) found that the onset and progression of OP is closely related to the metabolic regulation of the intestinal flora. The gut microbiota is one of the important pathogenic factors of OP and regulates the pathogenesis of OP through the microbial-gut metabolic-bone axis. Liu et al. (85) found that the effect of ethanol intake on the gut microbiota mainly increased the ratio of firmicutes and Bacteroidetes, which led to an increase in 5-hydroxytryptamine and inhibited the mineralization and proliferation of osteoblast-associated cells, thus affecting BMD. In addition, high lipids led to a significant increase in the relative abundance of bacteria, with a decrease in B. phenotypicus, B. actinomycetemcomitans and the bacteria of lipolysis and purine metabolism, which decrease the BMD (35). In addition, bone loss induced by salivary microbiota through the “oral-gut axis” in patients with periodontitis may be related to tryptophan metabolism and lipid degradation (88). These studies will contribute to a better understanding of the mechanisms and relationships between changes in osteoporotic metabolite levels and the gut microbiota, and how the gut microbiota is involved in and mediates the development and progression of OP, making gut microbiota regulation a new therapeutic strategy to promote healthy bone development.

Therefore, vitamin D, blood calcium, temperature, race, diabetes, osteoarthritis, atherosclerosis, gut microbiota and other factors both influence the metabolomic outcome of OP. In the future, it will be a meaningful research direction to further pay attention to and integrate various factors such as age, BMI, smoking and physical activity to explore the changes of metabolomics in OP.

## 5. Metabolomics for the development of OP therapeutics

**Figure 2:**
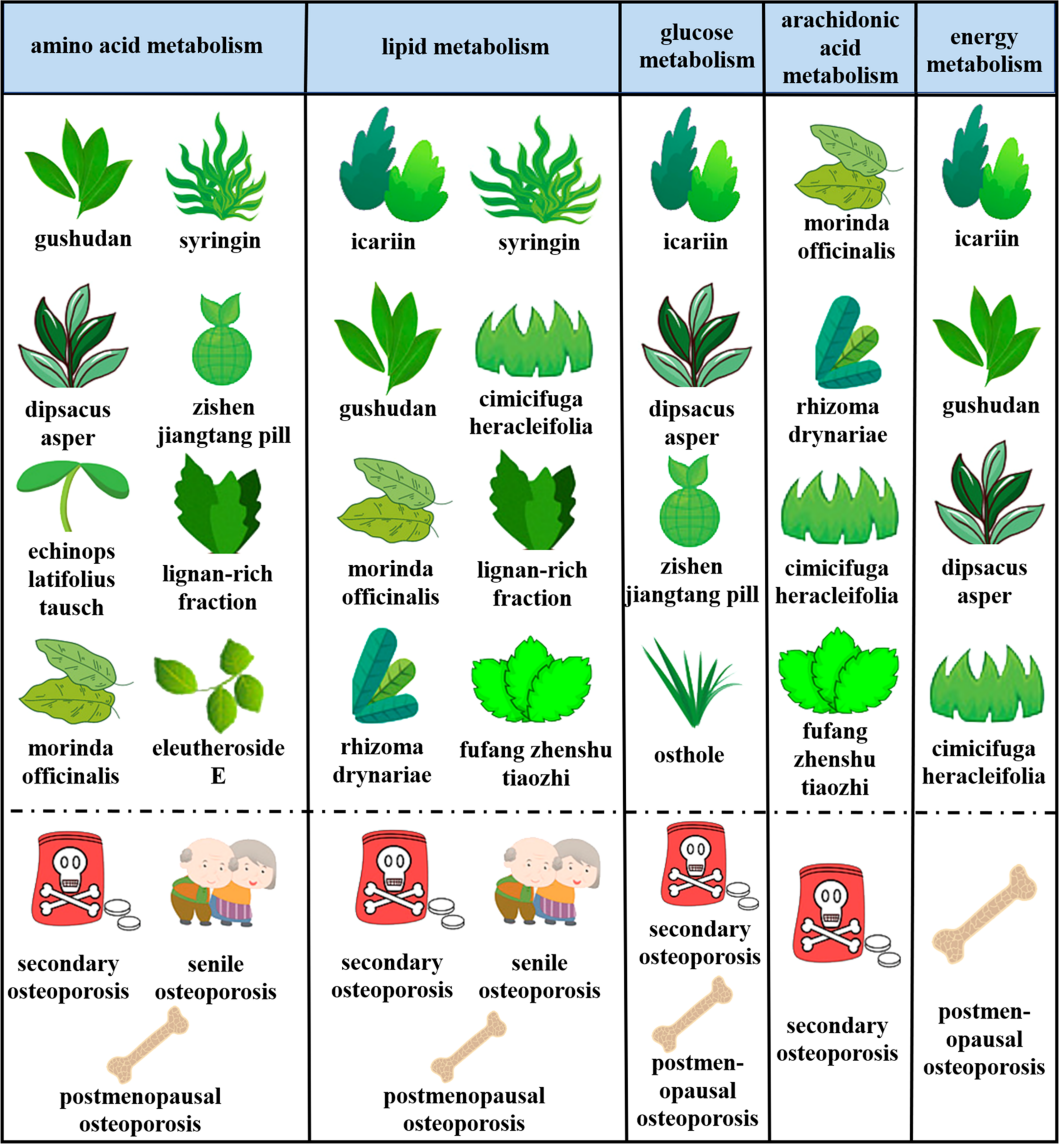
Metabolic pathways of different types of natural herbal action and therapeutic effects on different types of OP.

At present, the therapeutic mechanism of mature OP drugs such as alendronate sodium, teriparatide, calcitriol, etc. and the therapeutic effect of biomaterials have been systematically and deeply studied. There are also many studies and reviews summarized these drugs. In this review, we systematically summarized the OP treatment drugs that have been studied more in recent years, especially natural herbal medicine (**Figure 2**), and related extracts (**Table 3**), which have been found both protect bone from osteoporosis, but the mechanism need to be further explored. The introduction of metabolomics provides a good platform for the study of these drugs in regulating the biochemical metabolism of bone tissue, and can further explore the side effects, efficacy, and dose effect of their therapeutic methods. We provide a series of novel OP treatments to be developed and even laid a solid foundation for clinical transformation.

**Table 3:** Metabolomics for the development of OP therapeutics.

Type of drug	Therapeutic research subjects	Types of OP	Sample type	Analytical method	Significantly changed metabolites	Metabolic pathways targeted by drugs	References
Icariin	mouse, rat, chicken	postmenopausal OP, secondary OP	serum, bile, and urine	^1^H-NMR, UHPLCMS/MS, UPLC-QTOF/MS	**up-regulated:** alanine, creatine, taurine, glycine, β-glucose, uridine, palmitic acid, adrenic acid, fexofenadine, LysoPC (18:1) **down-regulated:** lactate, LysoPE (20:3)	glucose metabolism, lipid metabolism, energy metabolism, taurine metabolism	(89–92)
Gushudan	rat	secondary OP	serum, urine	UHPLC-MS, ^1^H-NMR	**up-regulated:** pyruvate, taurine, glycocholic acid, phenylalanine, creatine, valine, tryptophan, epoxyeicosatrienoic acid, hydroxyvaleric acid, benzoate **down-regulated:** lysoPC, creatinine, hippuric acid, lactic acid, leucine, citrate, hippurate, lndoxyl sulfate	lipid metabolism, amino acid metabolism, energy metabolism, purine metabolism	(93–96)
Dipsacus asper	rat	postmenopausal OP	serum, tissue	GC-MS	phenylalanine, serine, tyrosine, tryptophan biosynthesis, valine, isoleucine, biosynthesis, methane metabolism, glycine, threonine, galactose	amino acid metabolism, glucose metabolism and energy metabolism	(97)
Echinops latifolius Tausch	rat	postmenopausal OP	serum	UPLC-MS	**up-regulated:** proline, lysoPC, creatine, lysoPE, 9-cis-Retinoic acid, 4-Acetamidobutanoic acid, arginine, glycerophosphocholine, hydroxyprogesterone, N-acetylornithine **down-regulated:** 2-phenylethyl beta-D-glucopyranoside, anserine, quinaldic acid, pentahomomethionine,	amino acid metabolism, glycerophospholipid metabolism	(98)
Morinda officinalis	rat	secondary OP	serum	UHPLC-MS	**up-regulated:** 4-Pyridoxic acid, 11-dehydrocorticosterone **down-regulated:** L-valine, glycylproline, 4-Pyridoxic acid, valerylcarnitine, androsterone, N-phenylacetylaspartic acid, galactosylhydroxylysine, cortisol, docosapentaenoic acid, thromboxane A2, cortolone	amino acid metabolism, arachidonic acid metabolism, lipid metabolism	(99)
Rhizoma Drynariae	rat	secondary OP	serum	UPLC-MS	**up-regulated:** acrylic acid-2-acrylamido-2-methyl, cuscohygrine, santalyl phenylacetate, tetraHCA, N-oleoylethanolamine, **down-regulated:** indoxyl sulfate, narirutin, lysoPE, artocarpin, chenodeoxyglycocholic acid, L-palmitoylcarnitine, lysoPC, boviquinone, cholesterol sulfate	linoleic acid metabolism, glycerophospholipid metabolism and arachidonic acid metabolism	(100)
Syringin	mouse	postmenopausal OP	serum	UPLC-MS	**up-regulated:** 2-ketobutyric acid, cytosine, 3-methylhistidine, acetoacetic acid, normetanephrine, arachidonic acid, creatine, L-arginine, 3-methylglutaconic acid, lysoPC **down-regulated:** sarcosine, 3-aminoisobutanoic acid, dimethylglycine, d-ornithine, 2-aminoisobutyric acid, D-limonene	amino acid metabolism, lipid metabolism, Nucleic acid metabolism	(52)
Zishen Jiangtang Pill	rat	secondary OP	serum	^1^H-NMR	tryptophan, serine, 2-hydroxyisovalerate, anthosine, fumarate, uracil, creatine, acetate, threonine, 3-hydroxybutyrate, glutamate, formate, tyrosine, isoleucine, 2-oxoisocaproate	glucose metabolism, amino acid metabolism, nucleic acid metabolism	(101)
Cimicifuga heracleifolia	rat	postmenopausal OP	serum	GC-MS	**up-regulated:** oxalic acid, hydroxybutyric acid, glycine, L-phenylalanine, L-glutamine, D-glucose, stearic acid, arachidonic acid, myo-Inositol, palmitic acid, alpha-linolenic acid, cholesterol **down-regulated:** L-lactic acid, urea, creatinine, L-proline, L-glutamic acid,	lipid metabolism, amino acid metabolism, energy metabolism	(102)
Lignan-rich fraction	rat	postmenopausal OP	serum	UPLC-MS	**up-regulated:** uric acid, tryptophan, lysoPC (22:6), arachidonic acid, linoleic acid, oleic acid **down-regulated:** p-cresyl sulfate, sulfate metabolite, taurochenodeoxycholate, deoxycortisol/isomer, lysoPE (18:1)	lipid metabolism, amino acid metabolism	(103)
Fufang Zhenshu Tiaozhi	mouse	senile OP	serum	UPLC-MS	**up-regulated:** LPA, DG (36:3), PC (40:9), neuroprotectin D1 **down-regulated:** sphingosine 1-phosphate, lysoPE, arachidonic acid, fructose 1,6-bisphosphate, NADH, glycocholic acid, taurodeoxycholic acid	sphingomyelin metabolism, glycerophospholipid metabolism and arachidonic acid metabolism	(104)
Osthole	rat	postmenopausal OP	serum	UPLC-MS	**up-regulated:** 3-hydroxybutyric acid, taurocholic acid, LysoPC (15:0), citric acid, corticosterone, 8-HETE, Cer(d18:0/18:0), glutamine, uric acid **down-regulated:** lysine, linoleic acid, prostaglandin F2a, L-carnitine, glucose, arginine, ornithine, tryptophan, arachidonic acid, estriol	glucose metabolism, amino acid metabolism, energy metabolism, nucleotide metabolism, lipid metabolism	(105)
Eleutheroside E	rat	postmenopausal OP	serum	UPLC-MS	**up-regulated:** creatine, L-carnitine, creatinine, N-acetylhistidine, pyroglutamic acid, dopaquinone, N-a-acetyl-L-arginine, isoleucylproline, N-acetylvanilalanine, 5-acetamidovalerate, N-acetyl-L-tyrosine, estrone glucuronide, N-acetyl-L-phenylalanine **down-regulated:** kynurenic acid, cortolon, cortisol, dihydrocortisol, 18-hydroxycorticosterone, taurocholic acid, cholesterol, corticosterone, sulfate, 11-dehydrocorticosterone, cholic acid, 17-hydroxyprogesterone, tetrahydrocortisol, cholic acid glucuronide, prostaglandin G2	arachidonic acid metabolism, amino acid metabolism, glucose metabolism, lipid metabolism	(106)
Qing’e Pills	rat	postmenopausal OP	serum	UPLC-MS	sphinganine, 17a-Hydroxypregnenolone, arachidonic acid, alpha-Linolenic acid, corticosterone, docosahexaenoic acid, phytosphingosine, octadecadienoic acid,11-cis-Retinol, lysoPC, l-tryptophan, Tetrahydrocorticosterone, sphingosine1-phosphate, cholic acid, 1-lyso-2-arachidonoyl-phosphatidate, glycocholic acid	amino acid metabolism, fatty acid metabolism	(107)
Rehmanniae	rat	Secondary OP	urine	UPLC-MS	**up-regulated:** 4-Pyridoxic acid, 11-dehydrocorticosterone, corticosterone, 18-hydroxycorticosterone, **down-regulated:** benzoic acid, N-acetylproline, N-phenylacetylaspartic acid, androsterone/epiandrosterone, cortolone, lysoPA(i-14:0/0:0)	amino acid metabolism, Vitamin B6 metabolism, Steroid hormone biosynthesis, lipid metabolism	(108)
Achyranthes bidentata, chondroitin sulfate calcium	rat	postmenopausal OP	serum	UPLC-MS, LC-MS	**up-regulated:** glutarylcarnitine, lysoPC (18:1) and 9-cis-retinoic acid **down-regulated:** fatty acids, carbohydrates, dipeptides, carboxylic acids	glucose metabolism, amino acid metabolism, energy metabolism, lipid metabolism, nucleotide metabolism	(50, 109)
Estradiol	rat, osteoclasts	postmenopausal OP	skeletal muscle	UPLC-MS	**up-regulated:** phytosphingosine, palmiticamide, stearamide, alpha-aminobutyric acid, threonine, hydroxyproline, l-cystine **down-regulated:** lysoPC, lysoPE, stearamide, deoxycytidine, phospho-L-serine	purine metabolism, lipid metabolism, amino acid metabolism	(110, 111)
Lactobacillus	mouse, human	Secondary OP, Senile OP	stool, serum	UPLC-MS, LC–MS	**up-regulated:** lysoPC, L-alpha-Amino-1H-pyrrole-1-hexanoic acid, PE-NMe, N-oleoyl tyrosine, 15-HETE-VA, Lucidenic acid M, dihydropiperlonguminine **down-regulated:** reticulatamol, lsoleucyl-phenylalanine, N-acetyl-leukotriene E4, cysteine s-sulfate, fibrinopeptide A	lipid metabolism, fatty acid metabolism, amino acid metabolism	(112, 113)
Tocotrienol	human	postmenopausal OP	serum	LC-MS	**up-regulated:** betaine, 5-methylthioadenosine, methionine, gamma-glutamylleucine, gamma-glutamyltyrosine, N-acetylmethionine, N-acetylmethionine, cysteine sulfinic acid, S-adenosylhomocysteine, cystathionine, **down-regulated:** dimethylglycine, methionine sulfone	fatty acid metabolism, lipid metabolism, amino acid metabolism	(114)
Rubus coreanus Vinegar	rat	postmenopausal OP	serum	GC-MS, UPLC-MS	phenylalanine, tryptophan, butyric acid, lysoPC 22:6	amino acid metabolism, glucose metabolism	(115)
Bone marrow MSC	mouse	postmenopausal OP	femoral tissue	LC-MS	**up-regulated:** Acetylcholine chloride, Lipoxin B4 **down-regulated:** 3-Hydroxyanthranilic acid, l-Dopa, d-Xylitol, 5-l-Glutamyl-taurine, 5-l-Glutamyl-taurine, Melphalan	amino acid metabolism, lipid metabolism	(116)

### 5.1 Natural herbal medicine

#### 5.1.1 Natural compound herbal medicine

XianLingGuBaoJiaoNang was used to prevent and treat OP. However, there was no comprehensive metabolic profile of XianLingGuBaoJiaoNang. The results showed that cleavage and deglycosylation of glycosylated groups were the main metabolic pathways of various glycosides. Notably, amino acid binding was first found in the metabolism of pentene-flavonoid glycosides in the intestinal flora of rats (117).

The mechanism of Zishen Jiangtang Pill maintaining blood glucose and BMD is still unclear. These results indicate that Zishen Jiangtang Pill could effectively improve abnormal bone metabolism and glucose metabolism in diabetic OP, and was expected to be an effective alternative drug for the treatment of diabetic OP (101). Fufang Zhenshu Tiaozhi could treat hyperlipidemia and OP caused by glucocorticoid. Fufang Zhenshu Tiaozhi had a protective effect on senile OP, and its mechanism might be related to the interference of arachidonic acid metabolism, glycerophospholipin and sphingomyelin (104).

Xie et al (107) studied the effect of QingEWan on intestinal microflora in rats with OP. The levels of butyric acid, propionic acid and acetic acid were increased. In addition, QingEWan could regulate intestinal flora and improve OP.

#### 5.1.2 Natural single herbal medicine

Gushudan is a kind of traditional Chinese medicine preparation designed for secondary OP. Yuan et al (93) discussed the anti-OP effect of Gushudan on hormone-induced OP rats and its mechanism, and identified 40 different metabolites, mainly involving energy metabolism, amino acid metabolism, intestinal flora metabolism and fat metabolism. Using UPLC-MS technology of metabolomics, the overall therapeutic effect of Gushudan on secondary OP was effectively explored by detecting urine blood samples (94). By 1H NMR metabolomics method, 27 differential metabolites were found after Gushudan treatment. It was further proved that Gushudan may ultimately treat OP by changing these metabolites (95). Through a non-targeted metabolomic approach, Gushudan was further used to explore the therapeutic effect and related mechanism of secondary OP. The results showed that energy, fat, and amino acid metabolism play a huge role in this pathway (96). These correlation studies have systematically explored the therapeutic effect and metabolic mechanism of Gushudan. Metabolomics was also used to explore the mechanism of OP according to the above section. Several studies simultaneously found that the pathogenesis of OP is closely related to lipid metabolism, amino acid metabolism and energy metabolism. We summarized some key and jointly validated metabolites related with Gushudan in the **Table 3**. In combination with the regulation of Gushudan on metabolites of OP, we found that Gushudan significantly regulated taurine, creatine, Valine, tryptophan, and Leucine metabolites of OP (**Table 3**).

Tao et al (97) found that the Dipsacus asper treatment group had abnormal metabolic pathways. Dipsacus asper segment of liquor could treat and prevent OP by intervening energy metabolism, carbohydrate metabolism and amino acid metabolism in the body.

Wang et al (98) discussed the effects of Echinops latifolius Tausch on ovariectomized rats and the metabolic pathways involved in the changes in trabecular microstructure in OP. Echinops latifolius Tausch effected on bone trabecular microstructure of castrated rats may be related to intervention of glycerophospholipids.

Morinda officinalis and its chemical constituents could prevent OP caused by aging and estrogen deficiency. Metabolomics analysis showed that 37 different metabolites were present in the control group compared with the dexamethasone group, and 20 of them were significantly reversed after treatment with Morinda officinalis. Further Western blot analysis and metabolic pathway enrichment showed that Morinda officinalis prevented bone loss mainly through interference with arachidonic acid metabolism (99).

The mechanism of Rhizoma Drynariae anti-OP was still unclear. Using metabolomics, some potential biomarkers involving nine metabolic pathways were identified. These experimental results showed that Rhizoma Drynariae can prevent and treat OP by regulating the above-mentioned metabolic pathways, and provided a new theoretical basis for natural herbal medicines (100).

Cimicifuga heracleifolia was a traditional American herb that promises to counteract the ills of menopause. Serum metabolite composition was analyzed by serum metabolomics. The results showed that Cimicifuga heracleifolia has the effect of lowering blood lipid and anti-OP on climacteric syndrome. At the same time, its potential in improving metabolic disorders caused by postmenopausal OP was found (102).

Radices rehmanniae or dry Radices rehmanniae could prevent postmenopausal OP and senile OP. In metabolomics studies, 10 cases were significantly reversed after Radices rehmanniae treatment. These metabolites were mainly involved in amino acid metabolism, sex hormone regulation and steroid hormone biosynthesis. The mechanism of Radices rehmanniae action might be related to steroid hormone biosynthesis (108).

Rubus coreanus Vinegar had a good effect on postmenopausal OP. Of note, the Rubus coreanus Vinegar group had slightly increased levels of tryptophan, phenylalanine, lysophosphatidylcholine, glucose, and butyric acid compared with the postmenopausal OP group. Rubus coreanus Vinegar might be an effective natural substitute for prevention of postmenopausal OP (115).

#### 5.1.3 Natural herbal medicinal extracts

Icariin, the main component of icariin flavonoid glycoside, has been widely confirmed to have anti-OP effect. Some studies combined 1H NMR metabolomics and proteomics, and elucidated 8 metabolites in serum and 23 proteins in femur which were significantly changed (89). After a single oral administration of Epimedium, Cheng et al (90) determined the metabolites in rat urine, plasma, feces, and bile. The results also showed that the main metabolic pathways of icariin in rats were glycosylation and glycoaldehyde acidification after glycosylation. Pan et al (91) systematically analyzed the metabolomics characteristics of glucocorticoid-induced OP model rats. Huang et al (92) discussed the therapeutic effect and mechanism of icariin on low bone density in cage laying hens. Icariin mainly interfered with fat metabolism, taurine metabolism and pyrimidine metabolism of laying hens, resulting in increased BMD in old laying hens. Cobined these study of metabolomics applied to OP, we found that alanine, creatine, Taurine, Glycine, and β-glucose metabolites of the pathogenesis of OP were significantly regulated (**Table 3**).

Syringin had strong anti-OP activity, but the specific mechanism of its anti-OP was still unclear. High resolution mass spectrometry (MS) showed that metabolic pathways were closely related to catecholamine biosynthesis, butyric acid metabolism, glycine, tyrosine, methionine, and serine metabolism (52).

The part of Lignan-rich fraction in lignan was a traditional Chinese medicine used to treat bone diseases in China. Studies were conducted to identify potentially related metabolic pathways and metabolites. Studies have shown that Lignan-rich fraction can effectively restore amino acid-related tryptophan metabolism, lipids, and antioxidant systems (103).

Si et al (105) discussed the efficacy of osthole treatment. In the ovariectomized OP model, 28 metabolites were identified as biomarkers, some of which had significant regulatory effects.

In a study, the interventional effect of Eleutheroside E on postmenopausal OP was evaluated by analyzing the related metabolic network, potential biomarkers, and urinary metabolic profile of postmenopausal osteoporotic rats. This study explained the metabolic effects and pharmacological mechanisms of Eleutheroside E on postmenopausal OP (106).

Some studies have shown that Achyranthes polysaccharides can treat OP through various ways. This study evaluated the effect of Achyranthes bidentata polysaccharides on OP based on metabolomics analysis. Achyranthes bidentata polysaccharides had good potential in the treatment of OP (50). Metabolomics highly integrates the “top-down” integration strategy, and responds to various functional pathways and indicators through changes in metabolic pathways, networks, and end products, to understand the overall trend of system change. Therefore, using metabolomics methods, natural herbal formulations and extracts have received more extensive research and attention.

### 5.2 Hormone drugs

Estradiol is the main clinical drug for OP treatment. Wei et al. discussed metabolic changes in myogenic OP and the therapeutic effects of estradiol. The analysis showed that the changes of oxidative phosphorylation, tryptophan metabolism, glycerol phospholipid metabolism, thermogenesis, histidinine metabolism, arginine biosynthesis and purine metabolism were the most common pathogenic factors of myogenic OP (110). Liu et al. studied the response of osteoclast metabolites to estradiol using a metabolomics-based approach (111). Some 27 metabolites such as amino acids and lipid derivatives were significantly altered after estrogen action. The metabolomic pathway enrichment analysis showed that estrogen affects glycerophospholipid metabolism and played a therapeutic role in OP. Estradiol-induced changed in phosphatidylcholine-sterol acyltransferase activity, methyldialdehyde and malondialdehyde further affected glycerophospholipid metabolism. Studies have shown that estradiol is highly conditioned dependent on osteoclast metabolism.

### 5.3 Gut microbes

So far, fecal microbiota transplantation and probiotic supplementation have gradually attracted the attention of scholars as a new organ transplantation method in the alleviation and even treatment of OP. This approach aims to alter the abundance and composition of gut microbes in the recipient’s gut, thus affecting the metabolite levels in the body for the treatment of OP (118). Zhang et al (119) showed that gut microbiota treatment increased the levels of propionic and acetic acids, optimized the abundance and composition of the gut microbiota, inhibited the production of excess osteoclasts, and prevented bone loss in postmenopausal osteoporotic rats. Lactobacillus reassorts intestinal flora and alters metabolite composition, particularly lysophosphatidylcholine levels. Lactobacillus might be an effective and safe treatment strategy in some types of osteoporotic diseases (112). Lactic acid bacteria significantly reduced bone loss in older women with low bone density. Lactobacillus-regulated metabolites are involved in a variety of metabolic pathways, including acylcarnitine, peptide and lipid metabolism, as well as amino acid metabolism (113).

In addition, various drugs and bioactive substances could indirectly treat OP by directly modulating the abundance and composition of the intestinal microbiota. Calcium supplementation could increase the number of propionibacteria and immobile bacilli in the feces, thus affecting the concentration of short-chain fatty acids. Inulin could significantly increase the number of bifidobacteria and cocci, acting on the production of single-chain fatty acids and ultimately improving the mechanical strength, bone mineral content and BMD of the femur (120). lignan-rich induced high abundance of actinomycetes and restores microbial composition, which reduced abnormal lipid metabolism, prevents glucose tolerance, improves liver function, and reduced the risk of OP in castrated rats (121). Gushudan promoted the production of lactobacilli, which in turn acted on the production of lysine, acetate, and butyrate, ultimately acting as an anti-OP agent (122). Temperature exposure could reduce rumen bacteria and digestive cocci and increase lactic acid bacteria, lactobacilli and Lybacilaceae, thereby leading to changes in spermidine, spermine and polyamine levels and increasing bone strength (81). Qinga pill could change the composition of Firmicutes, Verumobacteria and Bacteroides in intestinal flora, and increase the content of butyric acid, propionic acid, and acetic acid in intestinal flora. The combination of anti-OP drugs and gut microbiota might be a new treatment for OP (107). In addition, Achyranthes achyranthes could regulate the levels of polyunsaturated fatty acids, lipids, glucose, and amino acids by acting on Escherichia coli, Roche, and anaerobic bacteria, thus exerting an anti-OP effect (123).

### 5.4 Other treatment strategies

Mao et al (80) investigated whether calcium supplementation can reduce bone loss in rats caused by calcium restriction and estrogen deficiency. The results of metabolomics analysis suggested that calcium supplementation was a metabolic pathway closely related to glycerophospholipid metabolism, and that the effect of calcium supplementation on OP might be due to increased estrogen levels, resulting in changes in metabolite levels, and ultimately increased BMD, thereby reducing bone degeneration.

In a population of postmenopausal women with OP, the effect of tocotrienols on metabolites was assessed using patient serum systems. When treated with tocotrienols, oxidative stress and inhibition of inflammation were significantly modulated resulting in a significant reduction in bone loss in patients (114).

Wang et al (116) discussed the efficacy of bone marrow mesenchymal stem cells in the treatment of OP in ovariectomized mice. Stem cell therapy could be intertransformed by glucuronic acid and pentose, metabase and taurine metabolism, and arachidonic acid metabolism. This study laied a foundation for the study of bone marrow mesenchymal stem cells as a treatment strategy for OP.

Chondroitin sulfate calcium complex was considered to have *in vitro* bone health activity. It was found that intervention with calcium chondroitin sulfate could alter fecal metabolite composition and intestinal microflora of castrated rats. Correlation analysis showed that certain intestinal flora was significantly associated with metabolite-rich and OP phenotypes (109).

As mentioned above, metabolomics has made a lot of progress in developing new treatments for OP. From the perspective of biochemical metabolism mechanism, in-depth research has been conducted on how various drugs such as Chinese herbal medicine, polysaccharides, hormones, and Lactobacillus act on metabolic reprogramming of the body and play a therapeutic role in OP (52, 89, 110, 113). Among a variety of Chinese herbal medicines, studies on the regulation of icariin and Gushudan on various OP metabolism are comprehensive and in-depth, involving fat metabolism, sugar metabolism, amino acid metabolism, pyrimidine metabolism, taurine metabolism and intestinal microflora disorders, etc (89, 93). Notably, we found that metabolites of the pathogenesis of OP, including Taurine, creatine, Valine, Tryptophan, Leucine, Alanine, creatine, Taurine, Glycine, and β-glucose were significantly regulated by icariin (90, 92). Therefore, further research on the therapeutic mechanisms of these two drugs should be more attached for clinical application.

## 6. Application of metabolomics in other researches of OP

The imbalance of bone resorption and bone formation caused by osteoclasts relatively active, leading to OP and accompanied by various metabolic disorders (124, 125). Therefore, specific changes of markers in various samples such as blood, tissue, and urine of patients with OP will reflect the characteristics of metabolic disorders of bone tissue, which can support the prevention and prediction of the disease (126, 127). Subtle changes in metabolites can be revealed by metabolomics, but these changes have not yet resulted in changes in bone density or structure. Furthermore, substances produced as the end products of metabolic activity are factors related to biological or metabolic states. Therefore, these specific metabolic markers are highly sensitive markers for the prevention and prediction of OP specific pathologic states.

### 6.1 Prevention

It is extremely important to explore some risk factors that reflect abnormal bone metabolism, and they can be used for early prevention of OP. For example, nutrition is closely related to BMD values in children and adulthood, therefore, rational nutritional intervention and treatment are crucial for the prevention of OP, which can further reduce the risk of osteoporotic fractures. Mangano et al (128) used an untargeted metabolomics approach to determine the biochemical factors driving the relationship between vegetable and fruit intake and the risk of OP. Vegetables and fruits can inhibit the synthesis pathway of lipid metabolites, and lead to increased concentrations of other metabolites in the body, thereby stimulating estrogen synthesis and slowing the progression of OP. Dietary prevention strategies with adequate intake of dark green leafy vegetables, berries, and melons are associated with significant improvements in OP development and progression in both men and women. Chau et al (129) studied metabolites associated with coffee and assessed their association with OP. The results showed that 12 serum metabolites were positively correlated with coffee intake, among which fenugreek, 3-hydroxypyridine sulfate and quinic acid had the strongest correlation. Of these metabolites, 11 were known to be involved in coffee intake, and six of them were involved in caffeine metabolism. In addition, explosion to some metals and heavy metals may also lead to bone metabolism disorders (130). 1 μM cadmium significantly affected the malate-aspartate and citric acid cycles, and 10 μM cadmium significantly affected the pyrimidine, alanine, glutamate, glucose-alanine, and citric acid cycles.

### 6.2 Prediction

Predicting OP is critical for people to maintain bone health and improve their overall quality of life. Existing series of risk factors are difficult to predict complicated OP risk. In recent years, through metabolomics technology, some studies have found that several types of metabolites can be used as potential predictive markers of OP. Kong et al (131) conducted a survey with an average follow-up of 9 years. In a community cohort study, high spermidine concentrations were associated with an increased risk of osteoporotic fractures. With further validation studies, spermidine baseline concentration may be a new alternative marker for OP associated brittle fractures. Therefore, spermidine and its related metabolites may be reliable predictors of OP. Untargeted metabolomics analysis was performed on serum samples from 32 normal controls and 32 patients with OP. Hyocholic acids plays an important role in the development of OP and may be a potential marker. Hyocholic acids may be a new target for predicting OP (132). OP is a chronic disease that manifests insidiously and is age-related, often not detected until after a fracture. Therefore, some studies have established a sensitive, accurate, and rapid predictive test method, and the related aminobutyric acid enantiomers and isomers are accurately detected and used to predict the progression of OP (133). Serum (R) -3-aminoiso-butyric acid and γ -aminobutyric acid were positively correlated with physical activity in young, lean women. This study opens new possibilities for aminobutyric acid as a potential predictor of OP.

## 7.Technological innovations in metabolomics and multi-omics integration to explore OP

With the rapid development of metabolomics in the field of OP, a series of traditional testing and analysis techniques and methods have drawbacks. Therefore, in recent years, technological innovations have been made in many aspects of metabolomics in the process of exploring OP diseases, and certain progress has been made. Furthermore, OP is a multi-factorial disease. Therefore, it is of great practical significance for OP to integrate metabolomics and multi-omics data for a comprehensive and systematic exploration. Wang et al (134) proposed a simple method to correlate the relative retention time of peaks in chromatograms with the intrinsic peaks and to assess their off-target performance using an LC-MS dataset obtained from plasma samples of rats with OP. The relative retention time method have fewer missing values, low peak intensity relative standard deviation, and good pattern recognition performance, which showed great potential in future metabolomics research. To improve the interpretability of the multiregional orthogonal projection model, they integrated targeted analyses of oxygen lipids, metabolomics, fatty acids, sphingolipids, and transcriptome. Clinical closure was also used for analysis. They identified OP genes associated with dysregulation of inhaled glucocorticoid metabolites, providing insights into the mechanism of BMD loss in asthma patients taking glucocorticoids. These results suggested that a combination of multi-block associative variable selection with multi-block orthogonal projection and interactive visualization techniques could generate hypotheses from multi-omics studies and inform biology (135). Yier et al (51) studied the anti-OP effects of oleanolic acid and used metabolomics methods to predict the mechanism of action. Oleanolic acid and methionine, cysteine metabolism, isoleucine, valine, phenylalanine, tryptophan, leucine and tyrosine biosynthesis, linoleic acid metabolism and other metabolic pathways were significantly affected. Using the new analytical platform, they will further understand the relationship between the therapeutic effect of oleanolic acid in improving OP and glucocorticoid-induced lipid metabolism, molecular transport, and metabolic changes in rats with dysglycemia.

The combination of metabolomic and metallomic methods to study OP is also one of the research hotspots in recent years. Tao et al (136) developed metabolomic and metallomic methods to explain the biochemical basis of the anti-OP effects of salt and raw achyranthes. Iron, manganese, zinc, glycine, ammonia cycle, alanine metabolism, arginine, galactose metabolism, copper, selenium, serine metabolism, lactose degradation, proline metabolism and urea cycle were increased. The combination of metabolomics and metallomics with pattern recognition and enrichment analysis of metabolites provided a useful tool for revealing the mechanism of action of traditional Chinese medicine. As a Chinese medicine prescription for clinical treatment of OP, it had the function of improving renal function and strengthening muscles and bones. Metabolic analysis identified 17 potential biomarkers associated with OP, including β -aminobutyric acid, glucose, arachidonic acid, and malic acid. Metallomic analysis showed that there were seven metal elements in rat kidney tissue: arsenic, iron, manganese, barium, molybdenum, selenium, and zinc. Metabolic pathways mainly included amino acid metabolism and glycolysis of the neurotransmitter. The combination of renal metabolomics and metallomics could effectively supplement the study of urine and blood metabolomics, which can not only effectively explore the pathogenesis of OP, but also explore the therapeutic mechanism of Gushudan on the disease (137).

Using bioinformatics methods, it was found that osteoblast differentiation was associated with an increased requirement for proline, and highlighted the strong demand of proline for osteoblast differentiation and bone formation (138). Kodriˇc et al. combined a variety of perspectives, including metabolomics, transcriptomics, proteomics, and genomics. The intersections were then analyzed to identify the common pathways or molecules that played an important role in OP prediction, prevention, diagnosis, or treatment (139). Combined with cell metabolomics and network biology analysis, fatty acid metabolism and galactose metabolism might be the main pathways affected by jujube side treatment (140). The pharmacological effects of naringin on OP remain unclear. Metabolomics analysis showed that 21 species were significantly regulated by naringin, including: pyruvate, amino acids, glycerophospholipids, polyunsaturated fatty acid metabolism, etc. Naringin was associated with changes in expression of 13 important protein targets by network predictive pharmacologic analysis. This revealed that the combination of network pharmacology and high-throughput metabolomics can further explore the metabolic mechanism (141). Heat exposure improves BMD and thus strength in castrated mice, primarily due to improved trabecular bone thickness, bone connection density, and bone volume. Comprehensive metabolomics and metagenomic analysis showed that temperature promoted bacterial polyamines biosynthesis, resulting in increased levels of total polyamines *in vivo*. The results of the study showed that the supplementation of spermidine enhanced bone density, and at the same time, the synthesis of polyamines in the body was inhibited (81). Tween-2 decoction is a Mongolian medicine for postmenopausal OP rats. Researchers combine untargeted metabolomics and network pharmacology and identified three key protein targets - hydroxysteroid dehydrogenase, cytochrome, and vitamin D receptors. Network pharmacology suggested that major changes in vitamin B6 metabolism were related to vitamin D receptor targets. Thus, Tween-2 decoction on postmenopausal OP rats might be related to down-regulation of vitamin D receptor (142).

In conclusion, the organic combination of metabolomics and bioinformatics, genetics, genomics, transcriptome, proteome, network biology, metagenomics, network pharmacology has made a lot of progress, realizing the systematic exploration of OP prevention, detection, and treatment (135, 137, 139, 141).

## 8. Challenges of clinical translation of metabolomics in OP research

Over the past decade, metabolomics has been increasingly used to identify biomarkers in disease and is currently considered a very powerful tool with great clinical translational potential (143). The development and utilization of metabolomics has enabled in-depth study of the metabolic characteristics of clinical disease, thereby optimizing disease mechanism exploration, prevention, prediction, and treatment monitoring. In theory, metabolomics can target metabolic therapy based on the metabolic dependence of OP to improve the specificity of clinical treatment (48, 49). From disease prediction to treatment, metabolomics opens new opportunities for comprehensive OP research. However, the clinical development and mass application of metabolomics still need to overcome some challenges and difficulties.

So far, non-targeted and targeted metabolomics have been widely used in OP disease mechanism and treatment research, especially natural Chinese herbal medicine. However, they need to overcome many obstacles and challenges before they can achieve clinical translation and widespread application (144). To overcome these drawbacks, a variety of complementary methods should be adopted to conduct metabolome research. At this time, more advanced instruments and platforms are required, which are difficult to achieve in both clinical and general laboratories. After obtaining a large amount of data, professional data processing and analysis software is often required, which requires certain professional skills of analysts, especially for non-targeted metabolomics. During data analysis, when the choice of the peak selection algorithm is changed, the data results will vary slightly. In addition, rational and rigorous experimental design is essential for analyzing large metabolomic datasets, which is also critical for the choice of statistical analysis (1, 57, 58). Therefore, targeting large-scale metabolomics research and clinical practice requires interdisciplinary collaboration and efforts of biologists, statisticians, and chemists. It is worth noting that, because metabolomics requires more high-end instrument platforms and specialized data processing algorithms, how to achieve standardization of clinical-level laboratory execution is crucial. In addition, the uniform standardization of institutional reporting and data analysis for metabolomics is another important challenge. Currently, most metabolomics studies produce relatively quantitative results. Absolute quantification is critical when normalizing across platforms. Previously, the Metabolomics Association had launched a standards initiative for a unified standard for metabolomics data communication. However, many published datasets still fall short of these criteria due to a lack of consensus among laboratories (145).

The results suggest that metabolomics can be used for prevention and diagnostic clinical treatment of OP. According to the results of a series of studies, these metabolites are indeed associated with OP. However, it remains to be determined whether these differential metabolites play a causal role in the pathogenic and pharmacological mechanisms of OP or are merely early manifestations of preclinical disease (57, 64). Therefore, patients with other diseases within 2 years of OP diagnosis should be excluded when exploring whether these differential metabolites play a direct causal role. In addition, the key point is that metabolomics generally uses plasma, urine, etc. of organisms for more overall evaluation and detection. At this time, it is difficult to distinguish between the metabolome changes caused by OP and those caused by other factors (146). Therefore, metabolomic analysis needs to effectively address these biological confounding effects in order to be better utilized in various studies of OP.

## 9. Conclusions

OP is a systemic metabolic disease. Metabolomics can effectively reveal the specific metabolic mechanisms of OP and the metabolic trajectories related to treatment response. In this review, the metabolomics of OP pathogenesis and metabolomics of natural herbal medicine are elaborated and summarized systematically. Some clinical translational studies have shown that metabolomics is a valuable tool to predict the therapeutic effect of osteoprotective agents and natural herbal medicine on OP recovery or to evaluate their side effects on normal bone function. In addition, metabolomics combined with gut microbiota studies have provided convincing evidence in the study of OP metabolism. In the future, the integration of gut microbiota and host may lead to more research breakthroughs and clinical application in the OP study. Therefore, metabolomics has good exploration value and clinical transformation prospect in many fields with many advantages in the study of OP.

However, the application of metabolomics in OP research still has some limitations. The multiple factors such as food intake, microbiota activity, the liver and muscle work together influence the levels of various metabolites. Therefore, which metabolites in urine, plasma, serum, bone tissue of OP patients can accurately reflect the development of OP in clinical application is still in the urgent exploration stage. In addition, the clinical transformation limitations of metabolomics are further reflected by the cellular heterogeneity. Thus, what metabolomics has in common with other omics approaches is that each technique alone does not capture a complete view of OP. Therefore, it might be helpful to combine metabolomics with other omics studies to further improve its selectivity and the effectiveness of clinical transformation. Further, the results of OP metabolomics may be affected by age, BMI, smoking, physical activity, gender, and other factors. At present, there is a lack of relevant targeted studies, and the extent and mechanism of the effects need to be clarified, which is a series direction for further investigation in the future. It is worth noting that the application of metabolomics technology in common clinical diseases is becoming more and more popular, but its application in the field of OP started late. At present, most of them stay in the stage of animal experiment, there are huge differences between animal experiment and clinical research, and there is still a long distance in clinical transformation.

## Author contributions

MC and KY conceived and revised the manuscript. ZZ and ZC wrote the manuscript. AC participated in the literature search and related data sorting during the revision. In addition, all authors contributed to the article and approved the submitted version.

## Funding

This work was supported by National Natural Science Foundation of China (82070902, 82272176), Shanghai Science and Technology Commission (19411963100), and Shanghai Jiao Tong University “Medical and Research” Program (ZH2018ZDA04).

## Conflict of interest

The authors declare that the research was conducted in the absence of any commercial or financial relationships that could be construed as a potential conflict of interest.

## Publisher’s note

All claims expressed in this article are solely those of the authors and do not necessarily represent those of their affiliated organizations, or those of the publisher, the editors and the reviewers. Any product that may be evaluated in this article, or claim that may be made by its manufacturer, is not guaranteed or endorsed by the publisher.
